# Emamectin Poisoning in Thailand: Clinical Characteristics and Outcomes

**DOI:** 10.3390/toxics12090668

**Published:** 2024-09-13

**Authors:** Satariya Trakulsrichai, Perapatn Sittiyuno, Phantakan Tansuwannarat, Achara Tongpoo

**Affiliations:** 1 Department of Emergency Medicine, Faculty of Medicine Ramathibodi Hospital, Mahidol University, Bangkok 10400, Thailand; perapatsit@gmail.com; 2 Ramathibodi Poison Center, Faculty of Medicine Ramathibodi Hospital, Mahidol University, Bangkok 10400, Thailand; phantakan.tans@gmail.com (P.T.); achara.ton@mahidol.ac.th (A.T.); 3 Emergency Department, Thung Song Hospital, Nakhon Si Thammarat 80110, Thailand; 4 Chakri Naruebodindra Medical Institute, Faculty of Medicine Ramathibodi Hospital, Mahidol University, Samut Prakan 10540, Thailand

**Keywords:** emamectin benzoate, avermectin, insecticide, toxicity, human

## Abstract

Emamectin benzoate (Emamectin) is a broad-spectrum insecticide. Current data regarding emamectin poisoning in humans are very limited. We performed a 10-year retrospective cross-sectional study (2011–2020) using data from the Ramathibodi Poison Center database to examine the clinical characteristics and outcomes in patients exposed to emamectin. Eighty-eight patients were included. Most of the patients were male (72.7%) and exposure was frequently oral (86.4%) and intentional (67.0%).Their mean age was 42.8 years. The clinical presentations included gastrointestinal tract symptoms (62.5%), neurological symptoms (27.3%) including seizures, respiratory symptoms (6.8%), and local effects (12.5%). At presentation, the majority of patients exhibited normal consciousness and vital signs. Eleven patients showed no obvious clinical effects. Initially, 15 patients had metabolic acidosis and 11 had hypokalemia. Overall, 46 and 52 patients were administered gastric lavage and activated charcoal, respectively. Most patients (78.4%) were hospitalized, with a median hospital stay of 40 h, and generally received supportive treatment. Eight patients were intubated for ventilator support and one received inotropic drugs. Most patients (90.9%) showed no or minor outcomes; however, two patients died. The presence of Glasgow Coma Scale (GCS) <15 differed significantly (*p* < 0.001) between patients with no or minor outcomes (n = 80) and those with moderate or fatal outcomes (n = 8). In conclusion, emamectin poisoning mainly caused no or minor clinical effects. A low GCS at presentation was associated with worse outcomes. Therefore, patients who present with low GCS should be closely observed, monitored, and properly managed during hospitalization.

## 1. Introduction

Emamectin benzoate (C_56_H_81_NO_15_) is an avermectin-class insecticide that was developed as a broad-spectrum insecticide [1,2]. Its mechanism of action for killing insects involves the stimulation of high-affinity GABA receptors, which increases the membrane permeability to chloride ions, leading to irreversible paralysis [1,2,3]. Insecticide products containing emamectin are available in various forms, including emulsifiable concentrates (ECs), water-soluble granules (SGs), water-dispersible granules (WGs), and liquids [4]. It is widely used in countries around the world, particularly United States and Canada [1,4]. It is also marketed and available in many countries in Asia, including Thailand.

The data for emamectin poisoning in humans are currently limited [4,5,6,7,8,9,10], and the available data are mainly derived from case series and case reports in Asia. Deaths have been reported for emamectin poisoning [7,8]. The major clinical manifestations are gastrointestinal (GI) tract and central nervous system (CNS) effects [5,6,7,8,9,10]. The documented clinical symptoms are sore throat, nausea, vomiting, abdominal pain, CNS depression, confusion, seizures, dyspnea, metabolic acidosis, and respiratory failure [4,5,6,7,8,9,10]. The data on emamectin pharmacokinetics in humans are also limited.

The Ramathibodi Poison Center (RPC) is a poison control center of a tertiary university hospital, the Faculty of Medicine Ramathibodi Hospital, Mahidol University, Bangkok, Thailand. Pesticide toxicity is a common poisoning that occurs in Thailand [11], and many pesticide poisoning cases are referred to the RPC [12,13]. 

A number of cases with a history of exposure to emamectin-containing insecticide products, which are mainly used in agriculture, have been referred to the RPC for consultation. Thus, this study was conducted to examine the clinical characteristics, management, and outcomes in cases of exposure to insecticide products containing emamectin in Thailand using the RPC database.

## 2. Materials and Methods

### 2.1. Study Design

A retrospective cross-sectional study was conducted using data from the RPC Toxic Exposure Surveillance System from January 2011 to December 2020. The primary objective was to describe the clinical characteristics and outcomes in humans exposed to products containing emamectin. The secondary objective was to identify factors associated with moderate to fatal outcomes.

The study was approved by the Institutional Ethics Committee of Ramathibodi Hospital Faculty of Medicine, Mahidol University (approval number COA. MURA 2021/432) and conducted following the Declaration of Helsinki regarding studies involving humans. The requirement for informed consent was waived because of the retrospective design of the research and the anonymized reporting of the confidential data obtained from the poison center database.

### 2.2. Study Setting and Population

The study setting was the RPC, which is based in a tertiary university hospital. Most consultations with the RPC were initiated by medical personnel. During the study period, the RPC undertook approximately 15,000–25,000 consultations annually. Follow-up telephone calls were conducted to collect patient data and progress data to enable further management recommendations. All data of cases were collected in the RPC Toxic Surveillance System database and were reviewed by senior specialists in poison information and clinical toxicologists.

The inclusion criteria were patients who were exposed to products containing emamectin and were referred to the RPC for consultation. The diagnosis of emamectin exposure was based on a history of exposure to emamectin-containing products determined by the ingredients listed on the bottle—in case the container was brought to the hospital—or the brand name, along with details of the ingredients provided by the patients. We excluded patients who co-ingested other pesticides, herbs, illicit drugs, or other chemicals. Patients who overdosed on pharmaceutical drugs were also excluded.

The collected data included demographic characteristics, exposure circumstances, amounts of exposure, durations from exposure to an emergency room (ER)/hospital visit, clinical manifestations, laboratory test results, hospital management, initial severities, and clinical outcomes. The severity of the poisoning was classified into five grades: none, minor, moderate, severe, and fatal. The definitions and terms used in the database were taken from the Poisoning Severity Score [14]. The amount of emamectin exposure was recorded in milliliters (mL). The ingested volume was estimated in mouthfuls (one mouthful = 25 mL) and cups/glasses (one cup/glass = 250 mL).

### 2.3. Statistical Analysis

The study data were collected using Excel software (Microsoft Office 2019, version 17, Redmond, WA, USA). STATA software (StataCorp, College Station, TX, USA) was used for the statistical analyses in this study. Continuous data are presented as the mean and standard deviation if normally distributed; otherwise, the median with minimum and maximum is presented. The frequency and percentage were evaluated for categorical data. Student’s *t*-test was performed to compare continuous data between groups if normally distributed and the Mann–Whitney U test was performed if non-normally distributed. Categorical data were analyzed for the between-group differences using either the chi-square test or Fisher’s exact test. Values of *p* < 0.05 were considered statistically significant.

### 2.4. Definitions

Hypotension was determined as systolic blood pressure <90 mmHg [15]. Tachycardia and bradycardia were defined as heartrate <60 and >100 beats per minute, respectively [16]. Fever was defined as body temperature >37.7 °C [17]. The normal vital signs for pediatric patients were determined based on their age [18].

Acute kidney injury was diagnosed in accordance with The Kidney Disease: Improving Global Outcomes clinical practice guidelines (Acute Kidney Injury Network criteria) [19]. Patients with no underlying diseases were assumed to have had normal kidney function before the exposure. Hypernatremia and hyponatremia were defined as serum sodium >145 and <135 mEq/L, respectively [20]. Hyperkalemia and hypokalemia were identified as serum potassium >5.0 and <3.5 mEq/L, respectively [20].

Metabolic acidosis was noted as arterial pH < 7.40 and serum bicarbonate <24 mEq/L or when noted in a patient’s records [21]. The range for a normal anion gap was considered as 7 ± 4 mEq/L [21]; therefore, a high anion gap was determined as ≥12 mEq/L.

## 3. Results

During the study period, a total of 88 patients with emamectin poisoning were identified and included in the study. The demographic data and exposure characteristics of the patients are summarized in Table 1. Emamectin-containing products involved in the exposure were identified in 43 patients. The forms were 5% WG (23 patients), 1.92% EC (12 patients), 2% microemulsion or ME (5 patients), and 5% SG (3 patients). Most patients were male (n = 64; 72.7%), and the predominant location was the central region of Thailand (n = 38; 43.2%). The mean age was 42.8 (range: 2–77) years. All patients were of Thai origin. Ten patients had underlying diseases, including diabetes, hypertension, small airway diseases, chronic kidney disease, psychiatric problems, and migraine. The main circumstances of exposure were intentional (n = 59; 67.0%) and accidental (n = 28; 31.8%), with no data available for the remaining patient. The major route of exposure was ingestion (n = 76; 86.4%), followed by inhalation (n = 11; 11.4%), dermal (n = 3; 1.1%), and ocular (n = 1; 1.1%). Three patients were exposed to two routes (inhalation and dermal routes, ingestion and dermal routes, and ingestion and inhalation).

The median duration from exposure to hospital visits was 1 h (range: 5 min to 48 h) (Table 1). Based on the data recorded at presentation, no patients had fever or hypotension. In total, 16 and 13 patients had tachycardia and tachypnea, respectively. Nine patients presented Glasgow Coma Scale (GCS) < 15. The most common clinical manifestations were GI tract (62.5%) and neurological (27.3%) symptoms (Table 2). Four patients presented with signs of moderate severity, including alterations in consciousness, seizure, and dyspnea. Another four patients presented with coma and their initial severity was classified as severe.

The laboratory results at presentation are presented in Table 3. Most of the patients presented with normal laboratory test results. Among 53 patients with recorded serum electrolyte levels at presentation, 11 had hypokalemia (serum potassium: 2.55–3.45 mEq/L), 3 had hyponatremia (serum sodium: 129–133 mEq/L), and 1 had hypernatremia (serum sodium: 149 mEq/L). A total of 15 patients had metabolic acidosis based on electrolyte analyses, comprising high anion gap metabolic acidosis in 11 and normal anion gap metabolic acidosis in 4.

Most patients (n = 69; 78.4%) were hospitalized, and the median hospital stay recorded was 40 h (range: 8 h to 19 days). There are no records of the length of hospital stay for five patients because they had recovered from poisoning conditions and were not followed up with until their discharge. These five patients prolonged their hospital stay for rehabilitation or treatment of complications such as infection; the poison center did not conduct a follow-up call until their discharge. Based on the clinical data during admission, we concluded that all of them survived. 

Most patients received only supportive care and achieved full recovery. Gastric lavage and activated charcoal were administered to 46 and 52 patients, respectively. One patient (1.1%) received inotropic drugs. Eight patients (9.1%) were intubated for ventilator support. Intermittent hemodialysis was performed on one patient (1.1%) who developed acute kidney injury during admission.

Most patients had minor (n = 55) or no (n = 25) clinical outcomes. Six patients had moderate outcomes, and two patients had fatal consequences. The clinical data for the eight patients with moderate and fatal outcomes are presented in Table 4. Patients 1–6 with moderate outcomes presented with neurological symptoms and required endotracheal intubation to protect their airway; they underwent extubation at a mean time of 16 (range: 11–24) hours after receiving supportive treatment. Some of these patients developed metabolic acidosis and improved following supportive treatment.

Patients 7 and 8, who presented with coma and developed systemic effects, eventually died. Patient 7 was a 41-year-old man with no previous medical history. He lived at home alone. A neighbor found him unconscious at his house after consuming an emamectin-containing product. In the ER of a rural hospital, he presented a GCS of 5 (E1V1M3). He was intubated and referred to the tertiary hospital. At the tertiary hospital, he received intravenous fluid. After admission, he developed hypotension and metabolic acidosis. An inotropic drug was initiated. He died rapidly within 8 h after admission. Patient 8 was a 73-year-old woman who intentionally ingested approximately 200 mL of an emamectin-containing product approximately 1 h before her arrival at the ER. After ingestion, she became unconscious. In the ER, she was comatose with GCS of 3 (E1V1M1) and immediately received treatment, including endotracheal intubation, gastric lavage, and administration of activated charcoal, before being referred to the tertiary hospital. The initial laboratory tests showed hypokalemia and metabolic acidosis. She received supportive care and was placed on a mechanical ventilator. She developed hypotension and bradycardia and finally died on the fourth day of admission.

The factors associated with moderate and fatal outcomes were analyzed by comparing the clinical manifestations between patients with no/minor and moderate/fatal outcomes (Table 5). GCS < 15 at the presentations was the only factor that differed significantly between these two groups.

## 4. Discussion

This study examined the data of patients with emamectin poisoning referred to the RPC and included 88 patients over a 10-year period. A previous retrospective study [10] recently presented the clinical data of 10 patients who experienced emamectin poisoning. That study reviewed the patients’ data from 31 August 2008 to 31 August 2023 and reported common symptoms, including sore throat, GI effects, corrosive injuries, dyspnea, and alteration of consciousness. The other case reports documented a single case each [5,6,7,8,9]. Given the paucity of data on emamectin poisoning in humans, with fewer than 20 cases reported in the English literature, this study represents one of the largest studies conducted on emamectin poisoning to date. Our findings provide valuable data and expand current knowledge on this type of poisoning, and the significance of this study is evident. Our patients were mainly referred from the central region of Thailand for consultations, reflecting the main agricultural area in Thailand. Pesticides are commonly used in that area.

Emamectin benzoate is a semi-synthetic avermectin insecticide that acts as a neurotoxin by interfering with the normal function of GABA. GABA functions as an inhibitory neurotransmitter in both vertebrates and invertebrates. Emamectin and other avermectins bind to GABA receptors, thereby increasing membrane permeability to chloride ions by opening chloride channels [3]. 

Neurotoxicity symptoms, such as tremors, ataxia, and tonic convulsions, have been documented in animal studies [2]. The increased toxicity of emamectin for invertebrates, compared to mammals, probably arises through its higher affinity for invertebrate GABA receptors compared to mammalian GABA receptors. Furthermore, emamectin benzoate cannot pass through the blood–brain barrier in mammals [3]. Nevertheless, toxicity has still been reported in humans after exposure to emamectin, especially in large amounts [5,6,7,8,9,10].

The clinical manifestations of emamectin poisoning observed in this study were GI tract, neurological, and respiratory symptoms. Our findings were similar to those in previous case reports and studies, wherein most patients had GI tract symptoms and neurological symptoms, including altered consciousness, myoclonic jerks, and seizures [5,8,9,10].

Based on the mechanism of action for emamectin in enhancing GABA activity, its toxicity commonly manifests as depression of the consciousness. Interestingly, however, seizures were also noted as toxicity symptoms in previous studies [2,6,8], as well as in the present study. Other studies have demonstrated that GABA agonists can exacerbate seizures in rodents and humans [22] and that drugs increasing GABA availability in the synaptic space tend to trigger seizures in animal models for certain forms of seizures [23].

Hypokalemia was observed in 11 patients (20.8% of 53 patients with recorded initial serum electrolyte levels), notably in 4 of the 8 patients with moderate to fatal outcomes. These findings were consistent with those in previous reports [7,10]. In one case series [10], 3 of 10 patients developed hypokalemia (2.8–3.4 mmol/L). The pathophysiology of the observed hypokalemia warrants further investigation. 

In this study, the two patients who died had symptoms of coma and hypotension. One died rapidly within 1 day of admission. Previous case reports have described fatal cases that exhibited severe metabolic acidosis and hypotension [7,8]. Although the mortality from emamectin poisoning is considered to be low, deaths do occur from this type of poisoning. Therefore, appropriate management and adequate supportive care are essential in patients with emamectin poisoning.

In this study, depression of the consciousness (GCS < 15) at presentation was significantly associated with moderate and fatal outcomes. Consequently, patients with alterations in consciousness should be admitted to hospital to be closely observed, attentively monitored, and properly managed. Prompt treatment should be initiated if patients develop other clinical abnormalities or clinically deteriorate during observation. 

There is no specific antidote or treatment for emamectin poisoning [1,2,10]. Supportive care, including proper management of complications that occur, may be the most suitable treatment for this type of poisoning.

The present study has some limitations. First, it may not represent the true incidence of poisoning and mortality rates because it is not compulsory to report exposure to emamectin-containing insecticides to the RPC. Second, the nature of the retrospective study design may have resulted in missing or incomplete data. The medical histories were obtained from the patients or their relatives, which they recognized and reported to the medical personnel. These histories may not be clearly or completely clarified. Finally, the diagnosis was primarily based on a history of exposure, with no laboratory confirmation of emamectin exposure.

## 5. Conclusions

In this study, emamectin-containing products commonly caused no or mild clinical effects. However, systemic effects occurred in some patients. GI tract symptoms were the predominant clinical manifestations. According to the mechanism of action of GABA agonists, neurological symptoms were periodically observed. The mortality from this type of poisoning was low. Good supportive care may be the most suitable treatment. A low GCS at presentation was associated with moderate and fatal outcomes, and thus, patients presenting with GCS < 15 should be closely observed and carefully monitored for clinical deterioration.

## Figures and Tables

**Table 1:** Demographic characteristics of all patients.

Characteristics (Number of Patients with Data Available)	n(%)
Sex	
Male	64 (72.7)
Female	24 (27.3)
Age in years, mean (SD)	42.8 (17.3)
Region	
Central	38 (43.2)
North	16 (18.2)
West	14 (15.9)
Northeast	11 (12.5)
East	7 (8.0)
South	2 (2.3)
Duration from exposure to hospital visit (minutes), median (min-max)	60 (5–2880)
Amount, median (milliliters) (min-max)	50 (1–300)

**Table 2:** Clinical manifestations of all patients at presentation.

Clinical Manifestations (Number of Patients with Data Available) *	n (%)
No obvious symptoms	11 (12.5)
Local symptoms - Gastrointestinal tract - Skin - Eyes	11 (12.5) 9 1 1
Gastrointestinal symptoms **	55 (62.5)
Neurologic symptoms ***	24 (27.3)
Respiratory symptoms ****	6 (6.8)

* Some patients had > 1 symptom; ** Including nausea and vomiting (n = 45), abdominal pain (n = 29), and diarrhea (n = 4); *** Including depressed consciousness (n = 15), dizziness (n = 9), and seizure (n = 2); **** Including dyspnea (n = 5), and tachypnea (n = 1).

**Table 3:** Laboratory findings of all patients at presentation.

Laboratory Findings (Number of Patients with Data Available)	Values (Mean ± SD)
Serum sodium (mEq/L), (53)	139 ± 3.7
Serum potassium (mEq/L), (53)	3.7 ± 0.4
Serum chloride (mEq/L), (53)	104 ± 4.06
Serum bicarbonate (mEq/L), (53)	22 ± 4.09
Anion gap (mEq/L), (23)	15 ± 4.5
Serum creatinine (mEq/L), (16)	0.97 ± 0.44

**Table 4:** Clinical characteristics and management of patients with moderate or fatal outcomes.

Characteristics	Patient 1	Patient 2	Patient 3	Patient 4	Patient 5	Patient 6	Patient 7 *	Patient 8 *
Patient characteristics								
Sex/Age (years)	Male/35	Male/44	Male/52	Male/74	Male/77	Male/30	Male/41	Female/73
Underlying disease	None	Psychiatric problem	None	None	None	None	None	None
Duration from exposure to hospital visit (minute)	120	300	N/A	30	60	60	180	60
Circumstances	Intentional	Intentional	Intentional	Intentional	Intentional	Intentional	Unknown	Intentional
Estimated amount of emamectin	30 mL	100 gm	N/A	100 mL	N/A	100 mL	N/A	200 mL
**Clinical manifestations at presentation**								
Local effects: oral mucosa irritation	No	No	No	No	No	No	No	No
Nausea/vomiting, abdominal pain, diarrhea	No, No, No	Yes, No, No	No, No, No	Yes, No, No	No, Yes, No	No, Yes, No	No, No, No	No, No, No
Alteration of consciousness, seizure	Yes, Yes	Yes, No	Yes, No	Yes, Yes	Yes, No	Yes, No	Yes, No	Yes, No
Dyspnea, secretion	No, No	No, No	No, No	No, No	Yes, Yes	No, No	No, Yes	No, No
**Systemic effects at presentation**								
HR (/minute), RR (/minute), BP (mmHg)	80,12, 120/80	97, 20, 142/103	70,18, 145/81	N/A, 20, 160/100	84,20, 110/70	96,18, 152/105	124, N/A, 170/110	50, N/A, 100/60
GCS	9	15	7	15	8	3	5	3
Metabolic acidosis	N/A	No	Yes	Yes	No	No	Yes	Yes
Acute kidney injury	N/A	No	No	N/A	No	No	N/A	N/A
BUN, Creatinine (mg/dL)	N/A	7, 0.8	21, 0.87	N/A	5, 0.66	5, 0.6	N/A	N/A
Serum potassium (mEq/L)	N/A	3.7	3.4	3.8	3.8	2.9	2.7	3.2
**Treatment**								
Oxygen therapy	Yes	Yes	Yes	Yes	Yes	Yes	Yes	Yes
Endotracheal intubation	Yes	Yes	Yes	Yes	Yes	Yes	Yes	Yes
Hemodialysis dialysis	No	Yes	No	No	No	No	No	No
**Complications during hospitalization**								
Pneumonia	No	Yes	No	No	Yes	No	No	No
**Hospital stays** (days)	5	19	2	3	N/A	2	0.5	4

Abbreviations: N/A, not applicable; mL, milliliters; Na, serum sodium; K, serum potassium; Cl, serum chloride, HCO_3_, serum bicarbonate; * Patients with fatal outcomes.

**Table 5:** Comparison of clinical features between patients who had no/minor outcomes and moderate/fatal outcomes.

Clinical Manifestations	Outcomes		*p*-Value
	No/Minor Outcomes (n = 80) (%)	Moderate/Severe/Fatal Outcomes (n = 8) (%)	
Male: female	58:22	6:2	1.000
Age (years), mean ± SD	41.7 ± 16.9	53.2 ± 18.9	0.072
Estimated ingestion amount (milliliters), median Route of exposure: ingestion	250 68 (85%)	70 8 (100%)	0.829 0.592
Initial signs and symptoms Gastrointestinal: abdominal pain, vomiting	53 (66.3%)	2 (25%)	0.340
Cardiovascular: tachycardia	15 (18.8%)	1 (12.5%)	1.000
Central nervous system: GCS < 15	3 (3.8%)	6 (75%)	<0.001
Respiratory: dyspnea	4 (85%)	2 (25%)	0.072
Length of hospital stay, median (Hours)	39 (12–118)	74 (8–456)	0.083

Abbreviation: GCS: Glasgow Coma Scale.

## Data Availability

The data of this article was presented at the 44th Congress of the European Association of Poison Centers and Clinical Toxicologists (EAPCCT), Munich, Germany, 28–31 May 2024, as a poster presentation with interim findings. The data are not available for public access because of patient privacy concerns but are available from the corresponding author upon reasonable request.
